# Digital work platform: Understanding platforms, workers, clients in a service relation

**DOI:** 10.3389/fsoc.2022.1075808

**Published:** 2023-01-04

**Authors:** Sofia Alexandra Cruz, Ana Gameiro

**Affiliations:** Faculty of Economics, University of Porto, Porto, Portugal

**Keywords:** digital work platform, algorithmic management, flexibility, work-life balance, service relation, platform-worker-client interrelationships

## Abstract

The rapid growth of digital economic activity had led to considerable scholarly interest in the phenomenon of platforms. Evidence shows how digital work platforms constitute one of the most relevant changes that have occurred in recent years and assume the condition of actors with an important presence in national and global work markets. However, these changes cannot be understood by focusing only on the work sphere, as the sphere of consumption is also central to this debate. In fact, the new ways of organizing, dividing and coordinating work on digital platforms are interconnected with specific modalities of consumption of the services made available by them. This article argues that a service relation approach allows an understanding of what is happening on digital work platforms, both in terms of the structural and conjunctural configurations of the interrelationships between platforms, workers and clients, as well as their social and economic consequences. This approach allows the analysis of the web of interdependencies between distinctive types of platforms, workers and clients, and to discuss how changes longitudinally within it are conditioned by the very transformations inherent to the platforms market. Thus, future research needs to explore the network of the voices of platforms, workers and clients in order to produce a robust analysis of these triangular relations as well as of the challenges regarding the differences and interconnections between algorithmic and human management.

## Introduction

Digital work platforms emerged in the mid-2000s, transforming work relations and leading to new ways of allocating tasks among workers (Eurofound, 2018; Crouch, 2019; Vallas and Schor, 2020; ILO, 2021; Rani and Furrer, 2021; Umar et al., 2021). The platform clients have the opportunity to disseminate the jobs to a large number of geographically dispersed workers inserted in the platform, i.e., the task is published to a crowd (Huws et al., 2016; ILO, 2018).

One of the dimensions often discussed in literature concerns the flexibility, temporal and spatial, provided by the platform work and its positive effects (Anwar and Graham, 2021). However, it is also emphasized in literature that the platform work provides working conditions considered precarious (De Stefano, 2016; Huws et al., 2016; Stewart and Stanford, 2017; Muntaner, 2018; Del Bono, 2019; Kahancová et al., 2020; Vallas and Schor, 2020; Tubaro and Casilli, 2022). Within this scope, one of the most salient characteristics is the inexistence of regulation in employment and in working conditions (Álvarez-Hernández and Pérez-Zapata, 2021), and this has been the major concern in scientific research recently.

However, the current debate on platform work has not yet sufficiently considered how platforms, based on algorithmic management, shape the interrelationships between workers and clients, leading workers to develop strategies that allow reframing the client as not simply an antagonist but also as a potential ally of workers within the interrelationships they establish with the platforms. This paper argues that these studies about platform work do not explore the relevance that worker-client interrelationships might have in determining the configuration of the triangular service relation between platforms, workers and clients.

The articulation between platforms, workers, clients involved in a service relation can be understood through the notion of three-way interest alliances (Leidner, 1996), allowing the exploration not only of a situational relation, but also structural patterns of interests and power relations crucial to deepen how platforms impact work and consumption (Kornberger et al., 2017; Culperer and Thelen, 2020). This notion is anchored in a triangular service relation—platform, client, worker—configured by intersubjective, institutional and spatio-temporal dimensions. Intersubjective, since they point to different modalities of physical and psychological engagement of each of the parties (Aroles et al., 2019). Institutional, as the platforms reflect the institutional environments within which they operate (Vallas and Schor, 2020). Spatio-temporal, for they are not reduced to episodic interrelationships, since often the contacts between the parties are reactivated in space and time through social relationships motivated by particular preferences or by the absence of choices (Kuhn and Maleki, 2017). Thus, the scope of this review is to argue that it is necessary an analytical framework that contemplates the triangular service relation between platforms, workers and clients. This is key to understand the articulations between the spheres of work and platform management, and the specific forms of consumption linked to digitalization and the widespread use of algorithms.

## Digital work platforms: Platforms, workers, clients

The evolution of the digital economy (Graham et al., 2020; Sasikumar and Sersia, 2020; Vallas and Schor, 2020) has been leading to the emergence of new business models and to new ways of organizing digital work and consumption (Culperer and Thelen, 2020; Alonso and Fernández Rodríguez, 2021; ILO, 2021; Rani and Furrer, 2021). The following sections review distinct evidences emerging from current scholarly discussion about platform work.

### Diversity of platforms, workers, clients

Digital work platforms function as intermediaries between workers and clients (Dunn, 2020), operating on a global scale (Bucher et al., 2021), and aim to combine supply and demand of goods and services by connecting clients with the professionals who offer their services (Del Bono, 2019; Howcroft and Bergvall-Kåreborn, 2019).

These platforms can be distinguished into two types, location-based and web-based platforms (Graham and Woodcock, 2018; ILO, 2018; Álvarez-Hernández et al., 2019; ILO, 2021). The former contemplates low-skilled tasks and require the physical presence of the worker when performing the work (De Stefano, 2016). In the web-based platforms, work is performed by geographically dispersed individuals who offer their services remotely (Howcroft and Bergvall-Kåreborn, 2019). These can take on two profiles: micro-task platforms, where works of short duration are performed, paid by the piece, which require less training and experience, without any type of direct contact between clients and professionals (Vallas and Schor, 2020) and freelance platforms that require higher qualifications, with the work being paid by the hour, and promote a more direct interaction between the client and the worker (Graham and Woodcock, 2018; ILO, 2021; Tubaro and Casilli, 2022).

The main characteristic of digital work platforms lies in the work being carried out digitally without the existence of the organizational structure of a company (Vallas and Schor, 2020). The work is coordinated, regulated and monitored through algorithms, and is disseminated to a crowd, thus replacing the functional authority structure of an enterprise, and without the worker often being aware of the end purpose of their work. The fact that these workers are classified as self-employed (Möhlmann and Zalmanson, 2017; Pesole et al., 2018; Graham et al., 2020), challenges the traditional patterns of work regulation (Stewart and Stanford, 2017). They are unable to benefit from protections recognized by the labor legislation (De Stefano, 2016; Stewart and Stanford, 2017; ILO, 2018; Graham et al., 2020), such as minimum wage, unemployment benefits, paid holidays, sick leave or maternity leave, among others (ILO, 2018). This condition becomes advantageous for platform clients to hire workforce, since they have no legal or social responsibility and avoid costs (De Stefano, 2016). Customers in turn are located in an interconnected environment of fast and competitive services, practicing an individualized consumption and following technological standards of the platforms (Alonso and Fernández Rodríguez, 2021). These consumers undertake fragmented consumption experiences associated with the execution of multiple and time-consuming tasks (Sadin, 2020).

Work mediated *via* digital platforms mandatorily implies the engagement of three parties, platforms, workers and clients (Florisson and Mandl, 2018), which are diverse among themselves and within themselves. It thus promotes a service relation that contributes to the detailed co-production of conditions of work and consumption (Briziarelli and Armano, 2020) that should be integrated and related in future research as more work and consumption are mediated through these platforms.

### Algorithmic management and platform work

Algorithmic management consists of a set of supervisory, administrative and control practices regulated by algorithms (Möhlmann and Zalmanson, 2017; Del Bono, 2019; Bucher et al., 2021). Through the implementation of these practices, workers' behaviors and performance are constantly monitored and evaluated, and algorithmic decisions are automatically made with scarce or no human intervention (Seaver, 2017; Jarrahi et al., 2020; Sasikumar and Sersia, 2020; Rani and Furrer, 2021). Customers, on the other hand, make their data available, adhere to various digital applications, in a mix of sovereignty and subordination (Sadin, 2020) and thus allow the platforms' algorithms to produce a large agglomeration of data generated by their digital footprint (Alonso and Fernández Rodríguez, 2021). This agglomeration of data not only serves to typify consumer profiles and reduce transaction costs between workers and customers, but it also promotes power relations between platforms, workers and customers that are instrumentalised according to coalitions of circumstantial interests (Bucher et al., 2021). These latter allow the platform to exert power over a considerable mass of people at the lowest possible cost and to impose the rationalism of standards, and classifications.

However, it should be stressed that such researches often do not sufficiently consider or even ignore the agency that workers can have in relating to algorithmic management (Anteby and Chan, 2018; Shapiro, 2018; Gandini, 2019; Gegenhuber et al., 2020; Kellogg et al., 2020). In fact, it is relevant to bring into the debate the argument that the exercise of power, while on the one hand it suggests asymmetric relations between agents associated with structures and practices of domination, on the other hand points to conditions for acting and therefore it is related to capacity and empowerment. It thus implies the simultaneous occurrence of relations of autonomy and dependence (Giddens, 1984). In the specific case under discussion, platform workers may adopt practices aimed at circumventing the platform algorithm and contribute to the co-construction of the algorithms' power through anticipatory compliance practices (Bucher et al., 2021). Thus, platform workers' agency is not only achieved outside of algorithmic management control, as evidenced by researches about the organization and mobilization of platform workers (Holts et al., 2021; Idowu and Elbanna, 2021) and self-organization strategies with the establishment of new associations of platform workers (Huws et al., 2019; López-Andreu, 2019), but also through its channels. Indeed, client sourced reputational metrics may expose workers to multiple forms of discrimination (Curchod et al., 2019), but workers can also establish connections with clients, for example, when they achieve client trust and are able to agree to allocate tasks directly without the need for platform intermediation (Jarrahi et al., 2020), ensuring benefits for both parties. To deepen this domain, the three-way interest alliances framework becomes fundamental as it shows the complex play of interests in this platform triangle between workers, clients and the platform itself, which may evidence different configurations.

### Flexibility and work-life balance

Literature is controversial regarding the question of the flexibility provided by digital work platforms, emphasizing two arguments. On the one hand, the benefits of platform work often mentioned are flexibility and autonomy (Del Bono, 2019; Wood et al., 2019; Álvarez-Hernández and Pérez-Zapata, 2021; Rani and Furrer, 2021), as workers are argued to be able to choose the amount of work and the tasks they wish to perform, as well as the time and place where they perform them (Rani and Dhir, 2020; Anwar and Graham, 2021), and the possibility of earning extra income (Barnes et al., 2015; Stewart and Stanford, 2017; Jabagi et al., 2019; Rani and Dhir, 2020). Furthermore, it fuels individuals' preferences to work from home (Forde et al., 2017), whether for health reasons, household burdens, the simple fact that they enjoy being in their homes (ILO, 2018) or for enabling the reduction of expenses and of time spent on public transport (Wood et al., 2019).

On the other hand, literature also mentions that this flexibility and autonomy may be likely to generate more uncertain, insecure and precarious work (Lehdonvirta, 2018; Wood et al., 2019; Sasikumar and Sersia, 2020; Sun et al., 2021). The absence of sufficient work on digital platforms is one of the factors that makes it impossible for professionals to perform tasks in greater quantity (ILO, 2021) and to be limited regarding the flexibility of their working hours, often implying unpredictable working hours or having to be available at non-regular times. Besides the lack of work, the absence of social benefits (Vallas and Schor, 2020; Masiero, 2021), job uncertainty and insecurity and financial instability are other drawbacks (De Stefano, 2016; Ashford et al., 2018; Dunn, 2020) associated with flexibility (Barnes et al., 2015; Howcroft and Bergvall-Kåreborn, 2019), mostly because workers are self-employed.

Moreover, the surplus of digital workforce also presents challenges, as workers feel pressured to please clients by reducing their salary proposals and performing work in reduced timeframes, and often allowing inappropriate behavior on their part, which can be a source of burnout (Bajwa et al., 2018). This is particularly evident when workers who are newly active on the platform become vulnerable in front of clients since they have no work experience and no profile on the platform. Thus, as a way to build their profiles and reputation on the platform, which is only doable by performing several works efficiently, they are often attracted to client schemes, accepting several works for which they are not paid after their completion (D'Cruz and Noronha, 2016). Moreover, the abundance of available workers also contributes to a more competitive work environment, as these professionals are easily replaced, mainly by those accepting a lower salary (Muntaner, 2018; Wood et al., 2019). In these circumstances, workers face working conditions that are considered adverse (De Stefano, 2016; Huws et al., 2016; Stewart and Stanford, 2017; Muntaner, 2018; Del Bono, 2019; Vallas and Schor, 2020; Tubaro and Casilli, 2022), which can have implications in terms of health (Eurofound, 2018; Muntaner, 2018; Wood et al., 2019; Anwar and Graham, 2021) and work-life balance (Álvarez-Hernández et al., 2019; Warren, 2021).

However, it is not only at this stage of entering the platform that such difficulties emerge but also in the stages that following it (Bajwa et al., 2018; Rani and Furrer, 2021). Thus, future research on how the different stages of the work cycle in the platform enhance distinct conditions and condition the interrelationships between platforms, workers and clients is fundamental. It is also important to address the degree of customer experience when contracting these digital services and how the conditions of production of a consumption process are configured. In this process customers are called to work at various stages of value creation (Dujarier, 2014), and to that extent may experience their work-consumption life balance in a considerably challenging way.

## Platforms-workers-clients service relation

Mapping the network of interrelationships that are established between the three parties of the service relation involving platforms, workers and clients becomes imperative in order to promote an integrated and holistic view about digital work platforms. In fact, it is important to explore to what extent the algorithmic management, which characterizes the functioning of the platform, differs according to the type of platforms and their clients (Vallas and Schor, 2020), and what are the implications arising from it for this triangular service relation. As recent evidence outlines, there are considerable differences in how platforms treat their workers and clients (Gandini, 2019; Veen et al., 2020; ILO, 2021) and how clients deal with platforms and workers (Kuhn and Maleki, 2017). However, such differences are interrelated and need further research.

As seen in previous section, platform work must be put into perspective in its triple nature constituted by platforms, workers, and clients. The platform constitutes a modality of economic activity that mediates the negotiation and the exchange between supply and demand, thus contributing to a new configuration of the work and consumption process. In this economic activity, value extraction lies in a new structural organization in which platforms remain powerful even when sharing dimensions of the work process related to the selection, control and evaluation of the workforce (Vallas and Schor, 2020), namely with their clients.

Although power often remains centralized in these platforms, it should not be forgotten that the exercise of power is relational and as such suggests a relationship of autonomy and interdependence. This means that platform workers may engage in practices aimed at dealing with the platform algorithm in the way that is most favorable to them, and to that extent contribute to the co-construction of the algorithms' power through their anticipatory compliance practices (Bucher et al., 2021) that they develop sometimes in conjunction with other workers and clients.

The professional life cycle on the platform needs to be framed in more depth in future researches. Indeed, it is not only newly arrived workers who face difficulties in managing their interrelationships with the platforms and clients (Popiel, 2017). If workers' practices change over time, such as various modalities of engagement in this type of work (Dunn, 2020), variations also occur in what can be referred to the customers' work-consumption life cycle, grouping different phases of consumption. Therefore, it is also urgent to problematise the extent to which such changes are conditioned by the transformations taking place in the platform market.

Considering only the interrelationships between platforms and workers prevents an apprehension of the complex interplay of interests underlying the configuration of the triangular service relation. Applying a service relation approach to platform work implies using a multidimensional framing to deal with the complexities and ambiguities existing within the interrelationships established between platforms, workers and clients. This approach highlights the central issue of the alignment of interests between, management, workers, service recipients and the fact that the degree of congruity of interests determines the configuration of the service relation, namely its degree of difficulty, the nature of the incentives underlying it, the routines and the resistances they generate, and the balance between the logics of efficiency and profitability with those of service quality and customer satisfaction (Leidner, 1996; Korczynski, 2009; Lopez, 2010). Following this approach, it is important to explore how routinisation works in this service relation and what outcomes it produces, particularly with regard to the extent of worker and clients acquiescence in algorithmic management of the platform.

The intersubjective, institutional and spatio-temporal dimensions of this triangular service relation warrant particular attention. The intersubjective dimensions suggest the importance of considering differences in culture, language and time zone, as well as the social nature of the relationship, which mostly involves virtual communication. The institutional dimensions, on the other hand, point to the relevance of considering that platforms are shaped by the institutional contexts where they operate, and therefore variables of economic, political and legislative nature of the different countries cannot be forgotten. Finally, spatio-temporal dimensions underline the fact that workers are hired globally, so that clients have access to the greatest possible diversity of potential workforce available, in a web of social relations guided by particular preferences or by the inexistence of alternatives.

Future research with a service relation approach it is required to understand the complexity of the triangle between platforms, workers, clients. It is also important to integrate a longitudinal strand that allows inferences to be made about how workers and customers relate to the platform over different life cycles of work and consumption, and how the configuration of these relations are shaped in space and time as the platform market itself evolves. Using a service relation approach highlights the ambivalences and power relations in the structuring of these triangular relations and helps in promoting a more balanced geometry in which platforms can provide a dignified, stable, and equitable environment for all parties involved in them.

## Author contributions

All authors listed have made a substantial, direct, and intellectual contribution to the work and approved it for publication.
